# Experiences from implementing value-based healthcare at a Swedish University Hospital – an longitudinal interview study

**DOI:** 10.1186/s12913-017-2104-8

**Published:** 2017-02-28

**Authors:** Kerstin Nilsson, Fredrik Bååthe, Annette Erichsen Andersson, Ewa Wikström, Mette Sandoff

**Affiliations:** 1 Institute of Health and Caring Sciences, Sahlgrenska Academy, University of Gothenburg, Box 457, 405 30 Göteborg, Sweden; 2 Sahlgrenska University Hospital, Göteborg, Sweden/Institute of Health and Caring Sciences, Sahlgrenska Academy, University of Gothenburg, Göteborg, Sweden; 3 Department of Business Administration, School of Business, Economics and Law, University of Gothenburg, Göteborg, Sweden

**Keywords:** Value-based healthcare, Implementation process, Qualitative study, Patient value, Health outcome measurement

## Abstract

**Background:**

Implementing the value-based healthcare concept (VBHC) is a growing management trend in Swedish healthcare organizations. The aim of this study is to explore how representatives of four pilot project teams experienced implementing VBHC in a large Swedish University Hospital over a period of 2 years. The project teams started their work in October 2013.

**Methods:**

An explorative and qualitative design was used, with interviews as the data collection method. All the participants in the four pilot project teams were individually interviewed three times, with interviews starting in March 2014 and ending in November 2015. All the interviews were transcribed and analyzed using qualitative analysis.

**Results:**

Value for the patients was experienced as the fundamental drive for implementing VBHC. However, multiple understandings of what value for patients’ means existed in parallel. The teams received guidance from consultants during the first 3 months. There were pros and cons to the consultant’s guidance. This period included intensive work identifying outcome measurements based on patients’ and professionals’ perspectives, with less interest devoted to measuring costs. The implementation process, which both gave and took energy, developed over time and included interventions. In due course it provided insights to the teams about the complexity of healthcare. The necessity of coordination, cooperation and working together inter-departmentally was critical.

**Conclusions:**

Healthcare organizations implementing VBHC will benefit from emphasizing value for patients, in line with the intrinsic drive in healthcare, as well as managing the process of implementation on the basis of understanding the complexities of healthcare. Paying attention to the patients’ voice is a most important concern and is also a key towards increased engagement from physicians and care providers for improvement work.

## Background

Value-based healthcare (VBHC), as a concept, has in recent years become established in Swedish healthcare organizations, in particular in hospitals [1]. In this study we explore how the representatives of four project teams experienced implementing VBHC at a large university hospital in their respective patient groups. The ideas behind VBHC were introduced by Porter and Teisberg in 2006. They built their framework, concept and practice on earlier management theories concerning competition and business strategy [2]. VBHC is based on three principles: creating value for the patients; basing the organization of medical practice on medical conditions and care cycles; and the measurement of medical outcomes and costs [2–6]. Even though these articles [2–6] are widely spread over the world, it has been questioned whether the original description of them fits the concept of VBHC as it is commonly understood today [7]. However in another empirical study about understanding VBHC it was found that participants understood the different parts of the concept but did not focus on all of them. Most of all they focused measuring medical outcome [1].

Porter and Lee suggested that creating value for patients and achieving success in implementing VBHC requires dedicated physicians and care providers within the organization [5]. They also suggested a set of independent steps to implement VBHC within the organization. The first step is to establish the common, indisputable goal of improving value for the patients. Value is defined as health outcomes attained per ‘dollar’ expended. They argued for a strategic agenda. The main component in this agenda is organizing care delivery into integrated practice units, which means organizing care around the patient’s medical condition instead of providing care in specialized departments. A further component is measuring outcomes of importance to the patients and their cost and comparing these results with others inside and outside the organization. They also suggested moving towards a bundled payment system; creating an integrated care delivery system; creating a geographically built up and excellent specialist health service; and investing in information technology platforms [5].

The Swedish payment system for healthcare differs from that suggested for VBHC, even though investigations and attempts to introduce bundle payment have been initiated [8]. Therefore, in this study, we do not focus on research concerning bundle payment systems in the overview of the literature. Apart from articles written by Porter and his colleagues about principles and strategies concerning VBHC, an increasing amount of discussion papers and some case studies have been published [9–14]. In one study using value-based management strategies for 4 years to improve care by employing best practice resulted in a reduction in readmissions, complications, and mortality, not to mention saving money [15]. In another study, an estimation of the value of care was made by assessing the impact of the harmonized implementation of processes throughout the period of surgical care instead of assessing just isolated outcomes. The researcher found improved outcomes and that the care delivered was more effective and thus of greater value [16].

In a longitudinal cross‐case comparison of implementing different management innovations in Swedish healthcare organizations [17] it was found that management ideas were adapted and developed gradually. Furthermore, support from senior management was necessary to monitor the type of innovation they were hoping to introduce although senior management should not be involved in its actual implementation [17]. A literature review [18] about investigation drivers and the challenges involved in implementing quality initiatives pointed out the importance of management, extended education in the new improvement before implementation, and a systematic implementation approach in order to succeed. According to Hellström and colleagues, implementing management innovation also needs to focus on which role the internal agents of change should have and their professional competence [19]. One example of management innovations in healthcare is process-oriented strategies. Conflicts over organizing principles and structures have been found to form obstacles to implementing process orientation [20]. When implementing process-oriented management strategies it has been found important to clarify process managers’ responsibility and work content [21]. Furthermore it was important to emphasize process managers’ basic requirements in order to do a good job [22].

There are differences between the Swedish healthcare system and systems in other countries, for example concerning effectivity [23, 24]. Thus, it is important to study how staff experience the process of implementing VBHC as described in Porter and Teisberg’s (2006) work ‘Redefining Health Care – Creating Value-Based Competition’. No studies of representatives of project teams’ experiences of implementing VBHC have been published to our knowledge up to date. This study aims to contribute to filling that gap.

## Method

### Aim

This study explores how the representatives of four pilot project teams experienced implementing VBHC over a period of 2 years in four different groups of patients at a large Swedish University Hospital.

### Design

An explorative and qualitative design was chosen in this study in order to understand participants’ experiences better with regard to implementing VBHC. Such a design is appropriate when little is known about a phenomenon [25].

### Setting

The starting point for the implementation of VBHC at the Swedish University Hospital in question was in October 2013 after that the hospital management team had decided to implement VBHC. This hospital, with about 2,000 beds distributed between 130 inpatient wards, provides highly specialized care and treatment to both children and adults. In 2013, the hospital provided healthcare in 107,000 inpatient care episodes, and in 180 outpatient wards with 1.2 million visits. The hospital employs about 16,700 employees. The implementation process started with four pilot projects for four different diagnostic groups. Each pilot project created a project team consisting of participants with different professional qualifications. Patient representatives were not invited to all of these team meetings, but were invited to participate now and then. The concept of VBHC was introduced by the hospital management team together with consultants from a consulting agency. The hospital management team had decided to engage external consultants to support the pilot teams and jump start the process to show fast progress. During the first 3 months, the consultants continued to support the project teams with procedural experience and knowledge. The process of implementation was expected to adhere to the following steps: mapping the group of patients; defining outcome measurements, and the process of measuring; collecting and analyzing data; developing and implementing improvement initiatives; and striving towards a continuous value-based work method. The defined outcome measurements were then listed on a scorecard, where each outcome was noted frequently, and thereby the outcomes could be followed and analyzed. Based on the analysis improvements could thereafter be undertaken.

### Participants

The members in respective team were appointed at department level. Each team consisted of five persons and they were all invited to participate in the study. One participant in each team had the managerial function of head of department. Participants’ profession and their function in the organization are presented in Table 1. Each team was composed differently, but every team had at least one healthcare developer, at least two physicians and one head of department (also a physician).

**Table 1:** Professionals represented in the teams and their function

Profession	Number
Senior Consultants (physicians)	9
Registered Nurses	3
Master’s Degree in Business Administration	3
Master of Engineering Degree	2
Psychologists	2
Occupational Therapist	1
Function	
Head of department	4
Healthcare developers	6
Working in different health professions	5
Controllers	3
Logistics	2

From the start, three of the pilot project teams had a physician leading the team and one team had a psychologist as team-leader. Due to employee turnover there is some small variation in age in each interview set. One participant did not want to participate at the third interview due to being too recently recruited and not yet involved in VBHC (see Table 2).

**Table 2:** Gender and age on each interview occasion

	Interview 1	Interview 2	Interview 3
Women	9	9	9
Men	11	11	10^a^
Mean age	47 (37–62)	47,3 (35–62)^b^	46,5 (34–62)

^a^One male participant had finished his employment and the one taking his place did not want to participate as he had not been involved in the VBHC process. Two more participants had finished their employment and were replaced by similar professionals

^b^One female and one male participant were on leave. The ones taking their place had the same profession but different ages

### Data collection

With permissions from the hospital management team and the medical director responsible for implementing VBHC at the hospital, each participant in the pilot project teams was contacted by telephone asking if he/she wished to participate. All 20 of those asked agreed. Thereafter they were informed about the study and that participation was at all times voluntary. This information was in writing but was repeated orally at the interview before it was started. A digital voice recorder was used for all 59 interviews and all were accomplished in a separate room at the participants’ workplace except one, which was carried out at a participant’s home (for the participant’s personal reasons). The first data collection period was set from March to April 2014, the second from November 2014 to January 2015 and the last was carried out between September and November 2015. The first interview varied between 37 and 64 min (mean 47), the second between 17 and 49 min (mean 31) and the third between 19 and 59 min (mean 33). All the interviews were transcribed verbatim by a secretary experienced in transcribing interviews.

### Data analysis

A tentative analysis was carried out directly after collecting each interview set. This was done to capture the main content in the interviews to give the participants the opportunity to provide feedback. This analysis also provides a written overview for use in subsequent interviews where the team could explore some questions in more depth. This first tentative analysis was guided by qualitative analysis [26] and it started with reading the interviews to gain a holistic picture of the material. The interviews were imported one after another into NVIVO 10 (software for qualitative analysis QSR International Pty Ltd) and that programme was then used for the further analysis. Based on the study aim, the interviews were read to capture meaning units, i.e., words and sentences that belonged together in terms of content. These meaning units were then condensed and labelled with a code. The next step in the analysis was to group the codes with their additional meaning units having similar content and to give these groups preliminary headings. This process was repeated for each data set. When all the interviews were analyzed up to this point, a thematic coding process followed where code groups from all three data sets were compared for similarities and differences. A phase of abstraction then followed. A comprehensive and interpretative analysis of the content continued by addressing questions to the material, whereby groups were reduced in number and expanded in content finally to form three themes with additional subthemes. The quotations are used in the results to illustrate the content in the themes and are marked with an identification code.

## Results

The three main themes that emerged through the analysis are related to the temporality of implementing VBHC: *getting started*, *being on the road*, and *being able to look ahead*. To these themes, additional subthemes are related describing variations identified in participants’ experiences of the process of implementing VBHC. An overview is given in Table 3.

**Table 3:** Overview of the result

Themes	Subthemes
Getting started	-Pros and cons of being guided by consultants
	-The process of identifying outcome measurements
	-Patients’ involvement
Being on the road	-Energy giver and thief
	-Getting stuck and later on regain renewed engagement
Being able to look forward	-Measurement as a means to improvement
	-Coordination between different developmental projects
	-Cooperation across borders

### Getting started

#### Pros and cons of being guided by consultants

The hospital management team had decided to provide these pilot projects with consultancy support. The participants appreciated the consultants’ promptness and efficiency and how they structured the work – for example mapping processes and choosing outcome measurements. The consultants initially directed the process of implementation of VBHC while participants contributed knowledge about their respective groups of patients as well as local prerequisites and possibilities:

Consultants from a consulting company gave us important support. They did not have any knowledge about our clinical practice, but they were very driving and controlling. (IP15)

The participants’ experiences of working with consultants were that they had contributed a lot of energy despite their participation generating an extreme amount of extra work. The consultants also contributed to retaining the focus on health outcomes when deciding which outcome measurements were relevant to use:

The consultants had a ‘watchdog’ role, or whatever you want to call it. When we proposed a measurement, they might say ‘but this is not a clinical outcome measurement, this is a process measurement’. (IP4)

In contrast to these positive experiences, participants also expressed the view that they were burdened by the pressure of time. Participants did not have time to anchor changes in work outside the pilot project team. It was more important to uphold the consultants’ time plan than actually to allow enough time for related health personnel to become involved and understand the concept. The speeded-up process involved participants having to reallocate time they would have used for clinical patient work since they did not receive any extra resources. The extra resources were only available to the consultants. The high tempo during the first 3 months deprived the participants of their own autonomy and they later lost their focus on the implementation of VBHC when the consultants had left. They were quite simply exhausted. The participants had no time to reflect over the concept and its adjustment to the actual setting; they just had to focus on doing what they had to do at each moment:

The time schedule created this fast tempo, which also meant that we lost the chance of understanding what we were really doing. (IP19)

Questions arose about the consultants’ working methods and their experience of healthcare conditions. According to participants’ experience, the consultants were more interested in demonstrating the results of their own work:

I feel that we have to chase results and try to prove things all the time; that they make their demands from the top down. (IP7)

#### The process of identifying outcome measurements

The process started with mapping the care processes for each respective group of patients. However, this seems to have been done as an obligatory duty in relation to the model decided for implementing VBHC, and not for any practical reason. The participants did not pay much attention to these mappings. One participant commented the process of mapping as follows:

As I experienced it, people wanted to round the mapping quickly to be able to say that now we’ve got a map. There was no focus on whether improvement proposals could be obtained via the map. That question never emerged, and then people do not remember having made a map. (IP17)

Instead the main focus when starting up was to identify relevant outcome measurements. Participants experienced they were being rushed to find out which measurements would be suitable when creating a scorecard for each patient group. Process measurements – for example cost per days of care and hospital stay – had previously been used to evaluate healthcare. Now participants had to think differently and focus on what created value for the patient. Then they had to find out which measurements should be used. When participants concentrated on the patients’ perspective they were sometimes prevented from choosing what they really wanted to measure due to lack of data or opportunity to search for statistics in the hospital’s IT system. These difficulties caused a lot of discussions and gave personnel the feeling that they had to choose accessible and existing measurements:

We bandied ideas back and forth, also with the patients in the group. And of course from the beginning there were a lot of things that we wanted to measure. Sometimes there were things that we would have liked to measure, but there were no statistics, so we just had to give up. (IP11)

Sometimes but not always, the patient representatives participated in these discussions, and according to participants they contributed with valuable opinions. A restricting factor in choosing outcome measurements was that participants were more or less referred to using data from National Quality Registries. Even if there were possibilities to influence the set of variables in these registers this was a lengthy and time-consuming procedure. On the one hand participants experienced the National Quality Registries as valuable and necessary to catch up on medical outcome measurements; on the other they found this data useless when it came to managing healthcare in the short run:

Quality of life is a very interesting parameter but we can’t use that as the deciding factor since there’s up to a 6-year time lag. So it doesn’t help us very much in the short term. (IP3)

Even in the startup phase, the participants paid attention to difficulties in measuring costs. Existing measurements were on an aggregated and standard size level. These difficulties could not be solved by the participants themselves. Therefore these questions were addressed to the hospital management level and the participants in the pilot project teams continued to measure costs as they had done before.

#### Patients’ involvement

The participants appreciated the focus on value for the patient. To strengthen the focus on value for patients all pilot project teams invited representatives from current patient associations to participate in some team meetings. The participants explained how important the representatives’ contributions were since the participants were aware that they did not always understand the patients’ point of view:

We as professionals think we know what value is for the patients but that competence we really don’t always have … therefore we invited patient representatives. (IP9)

Even if it was impossible to make use of all the patient representatives’ opinions and experiences, participants were proud of their cooperation with the representatives as this contributed to the legitimacy of their implementation work. The representatives participated at meetings, listened to the discussion and then took these discussions with them back to the patient association to anchor opinions and suggestions by their members. The participants found that there was sometimes a discrepancy between patients’ experiences of value and how participants thought they delivered value. They understood that they had taken things for granted and not always seriously evaluated the delivery of care in relation to value for patients:

We were influenced by comments from the patient representatives. There were for example concrete views on patient education and on how to measure what happens to patients, how patients are doing and so on. Then of course we couldn’t use all their ideas, but they have certainly had an impact on the work. (IP14)

On the other hand, the participants thought it might have been difficult to be a patient representative, and that they were sometimes left behind in the discussions. So it was important to invite representatives into the discussion and also to choose relevant questions for them to discuss. Participants also mentioned that patient representatives were invited to participate since this was a component of the concept. Participants emphasized the importance of patient representatives, but pointed out at the same time that health professionals always take their point of departure in their own fundamental values:

But of course we do have, so to speak, a conception of what we should do for the patient. We must not cause damage and we should preferably cure and relieve them and so it must be reasonable to believe that we *know* what we will achieve. (IP10)

Participants also took up the importance of research results as a basis for choice of outcome measurements. Health professionals ‘with quite a lot of research skills’ (IP9) were seen as guarantors for critical thinking in the teams. These critical thinkers were expected to pay attention to research when choosing outcome measurements. An example mentioned was research showing that ‘patients are not as satisfied with the result of the operation as we think’ (IP8). That kind of knowledge was used to motivate the participation of the patient representatives.

### Being on the road

#### Energy giver and thief

After a year some participants considered that they were no longer in the startup phase, but that their implementation of VBHC was more and more incorporated into their daily work. However not all participants had this experience. Other participants were still continuing with their scorecards and discussions about which outcome measurements were the best. And some even talked about having taken a break in their implementation work. However, whatever the status of their implementation works, participants still experienced the concept of VBHC as commendable. Working in accordance with VBHC intentions, especially creating value for the patients influenced participants positively and gave them the energy to make an extra effort during their implementation work. Participants described how tired they were of the earlier one-sided focus on costs and ‘budget in balance’. They were satisfied with VBHC as a method to increase quality in care provision and were convinced that quality would be profitable in the long run. The focus on value for the patient, emphasized by the hospital management team, contributed to their feelings of ‘enthusiasm for the concept and strong engagement in implementation work’ (IP2). The fact that problems in relation to care delivery had been identified and improvements had started up also gave new energy to continue with implementation:

I feel filled with enthusiasm when talking about VBHC, making these changes in order to increase the value of what we do for patients, instead of focusing solely on money. (IP13)

At the same time, warning voices were raised. Participants worried that this might be a management trend, coming and going like other trends, and they thought the widespread interest in VBHC was like a kind of revivalism. They were anxious about whether they would be able to maintain their enthusiasm and engagement in the long run:

Actually, we’ve really been practicing VBHC from the start, but we’ve been worried about if we would be able to keep it up, but so far we’ve managed to keep going. (IP1)

The participants talked about energy being drained by the lack of IT systems supporting VBHC, most of all in relation to measuring costs, but also in relation to health outcome measurements. Participants had difficulties in easily accessing information in different IT systems within the hospital organization. As health professionals it was frustrating not being able to influence these systems:

IT issues have been a complicating factor that we really do not have any control over. I feel that the systems we have are not designed for use in these kinds of health outcome measurements. (IP4)

According to participants, another energy-draining factor was that the project leader did not always have the authority to make decisions. The importance of this mandate appeared as implementation work progressed. Factors decreasing value for the patient had been identified in the organization and management authority was needed to solve these problems. For example, if the need arose to change the working methods of a group of staff, this could only be met through a managerial decision:

Some questions hung in the air, and the team leader was sitting there with staff he was not a head of. He had no mandate to decide anything; they had their own manager. (IP11)

#### Getting stuck and later on regain renewed engagement

When the consultants concluded their consultancy it was difficult for the participants to retain their focus. Even if the implementation work was important and prioritized work, participants were divided between different obligations. They were even more unfocused when the tasks they had initially pushed aside called for their attention. These hindrances contributed to decreasing engagement in carrying the process forward. Another factor influencing the possibility to keep up the focus on VBHC was the degree of staff stability, not just within the project teams, but also in different working groups. The project teams were mainly intact with respect to members, but there were variations in the members’ fields of responsibility. The ones who were project leaders were especially important for continued stability; changing the team leader involved reducing the speed of the implementation work:

There is the risk we’ll lose momentum if we have to change leaders along the way. (IP9)

During implementation work, participants identified and expressed frustration about how healthcare was organized in departments and how financial responsibility on department level obstructed cooperation between departments; cooperation necessary to create value for the patients. This organizational structure was frustrating as this contributed to difficulties in tracking and following patients during the course of the disease when they crossed boundaries between departments. This was also a reason participants got stuck in their implementation work:

We’re not very used to working under both matrix and linear management/…/We have a strong linear organization; people get confused when we have to start working between silos according to the principle of value for the patients. (IP9)

Engagement for VBHC also decreased when participants did not see any actual activity or result of their implementation work. Results that would have helped were ones that solved the problems of delivering care and cure in time without sending cancellations to patients. Being forced to make cancellations caused frustration among participants. They then lost their confidence in working with VBHC and found it meaningless trying to make smaller changes in the process when the great problem was lack of capacity. This frustration contributed to one pilot project team interrupting the implementation process:

Our engagement for VBHC is fluctuating, not least at the present time, when it’s low because we’re not carrying on with any VBHC implementation work … our only focus is to try to do our job, i.e. to care for and cure patients. (IP3)

At the same time other participants experienced renewed engagement. When they had passed the period when a lot of energy was put into identifying outcome measurements, they could instead start analyzing outcome measurements and working on concrete improvement initiatives:

To leave that, the abstract business of just looking at measurements, so to speak, and instead to try and understand and find good aspects to work on and develop in practice, now that’s a good thing to do. (IP7)

Even if the process of implementation did not run smoothly for all the project teams, they found it valuable to be able to evaluate their processes once again to give themselves a clear picture. They also toned down their expectations of quick results as they understood this was more about changing culture:

Actually this work is a cultural journey. One can’t hope for rapid change just by writing a memorandum (PM), or that things will sort themselves out – they won’t, but we are well on the way. (IP11)

### Being able to look forward

#### Measurement as a means to improvement

Reliable data is essential for being able to measure health outcomes. Therefore, the first step was to create requirements for acquiring reliable data to analyze. Efforts were made to increase the coverage of registers, to facilitate the development of new routines and support systems for participants when registering patient data:

We’ve increased the coverage. This is one of the effects of VBHC. It is actually a first step towards VBHC. (IP4)

The implementation of VBHC also speeded up the construction of local quality registers with the potential to become national ones, for groups of patients in need of treatment at another department:

…she has developed the registry, and I think it will become a national one. It will be used both as a basis for discussion with the patient, and will at the same time be a warehouse for register data, and that is very positive. (IP7)

At one department connected to one of the groups of patients, the department had for several years developed a local care quality register. This register was now used to measure and improve health outcomes. One example was measuring patients’ falls. This measurement (among others) was followed up by the VBHC team. When increases in falls were observed, the team increased fall prevention and could promptly see the effects of their preventative work. Such concrete examples contributed to spreading knowledge and engagement thus creating value for the patients. That, in turn, impacted the culture, for example, evolving what was considered important for a senior physician to care about:

I’ve actually been through an incredible change concerning interest in falls. The senior consultant asked me for the first time ‘what about falls?’ (IP20)

Participants said they improved their capacity to follow patients’ health outcomes as well as the routines and processes to promote them. The VBHC concept had taught not only the participants, but also other health professionals working in the departments involved, the importance of measurements in evaluating care. The participants no longer initiated any changes whatsoever in routines, as they had earlier, unless they had measurements as their point of departure. The participants found that measuring different variables contributed to the possibility of identifying not only what they needed to do better, but also what they did wrong. Detecting divergences in the scorecards led to improvements in care processes as well as the development of new procedures.

#### Coordination between different developmental projects

Participants said they had worked on improvements before starting on VBHC implementation, but that implementing VBHC implied working in a more systematic way. They did not always finalize ongoing improvements separately, but instead incorporated them into VBHC. One participant said ‘people think about what are we doing and then squeeze that into the model, even if it is not VBHC’ (IP16). This was done differently depending on the status of the improvements and existing working methods. Incorporating existing improvements in the VBHC system also contributed to the satisfaction of having improvements to demonstrate. Some departments were more prepared than others to start monitoring health outcomes:

Much of what has happened during this period we would have done irrespective of VBHC, because we were already thinking and working this way. What VBHC has contributed is better focus. We now have much greater ability to control and monitor and react. So VBHC comes out on top, so to speak. (IP8)

Combining existing improvements with VBHC was seen as an effective way of working since the improvements could reinforce each other. Participants mention examples of parallel improvements. One example was continuing to implement production planning. This was already implemented in the department’s outpatient wards and continued at inpatients wards parallel to implementing VBHC:

Whatever the improvement, if you lift it in it will become part of VBHC. There’s really nothing to say that an improvement must use a certain method. (IP6)

#### Cooperation across boundaries

As the implementation of VBHC advanced and outcome measurements were obtained and analyzed, participants noted need of cooperation with other departments. Despite cooperation developed as a result of VBHC still being in its infancy, participants gave several examples. These might involve cooperation with service departments such as radiology or pathology or with other care institutions in the care chain. One example given was deepened cooperation between departments providing care for a specific group of patients. The two departments involved already enjoyed good and extended cooperation but were under different management and had different budgets. VBHC had made organizational improvement imperative in order to facilitate further cooperation between professionals at these departments. This increased cooperation made it easier to obtain outcome measurements and to carry out patient follow-ups. Increased cooperation also increased understanding of different conditions in each department and of conditions for different patient subgroups:

After dialogue between the heads of department, there has been great progress in our team, which from the beginning focused on patients undergoing surgery. Now we have a representative from the other department in our team. So now we have much better preconditions for working with the whole group of patients. (IP7)

VBHC also increased awareness of cooperation between inpatient and outpatient care. This kind of cooperation contributed to increased accessibility for the patients to receive care at the right care level. To facilitate mobility within the care chain, for example, participants discussed videoconferences between professionals in out- and inpatient care, to speed up the process: ‘we are using videoconferences between outpatient and inpatient care to increase accessibility’ (IP14). Other initiatives mentioned were cooperation with primary care and community care respectively. This kind of initiative was introduced by team leaders with the immediate aim of decreasing length of care in hospital. The overall aim was to decrease hospital care-related complications, thus saving costs.

## Discussion

Creating value *for* the patients is, according to Porter and colleagues, the overall goal when working with the concept of VBHC [2–6]. Most of all, participants associated the implementation of VBHC with value for the patients and a focus on how to measure health outcomes. This emphasis on value for the patients is confirmed in other studies [1, 7] and may be understood as health professionals’ intrinsic motivation [27] to use their competence to care for, cure and relieve suffering for the patients. Participants’ positive response towards creating value for the patient may also be explained and understood in relation to NPM (New Public Management). NPM is a management model that has for more than two decades been implemented in this setting. It focuses on efficiency, the internal control of financial aspects and market-like arrangements between units in the organization [28, 29]. NPM have been criticized due to its one-sided dominance of quantified values [30].

For whatever reason, less attention was paid by health professionals to measuring costs. Accordingly, the results mainly emphasize two of the aspects in the concept of VBHC, i.e., value for the patients, and measuring health outcomes. It is therefore debatable whether or not VBHC was really implemented or whether it was just an inspiring concept. On the other hand that is mostly a definitional debate with limited clinical impact. As it was, it reintroduced value for patients as the overarching objective for what healthcare is all about. It also reignited the health professionals’ sense of engagement, especially that of the physicians, in the continuous journey towards the further development of quality of care.

The implementation process initiated by the Hospital Director both demanded a lot of energy and simultaneously gave energy when the participants had adjusted the concept to local practice and thus experienced improved patient care. In a study concerning staff’s responses to paradoxes experienced in organizations, it was found that Human Resources staff developed a skill that enabled them to translate top-down strategic decisions to fit different local conditions; a skill that was important for implementation [31]. In line with this, the participants in our study also seem to have been able to translate the intention of creating value for the patients to fit their previous professional understanding of what actually does constitute value for the patients. The process of implementation was not linear but more of an evolution, similar to the way described by Øvretveit and colleagues [17]. Most participants in our study lost momentum after the three first months when they were guided by consultants, but after some rest most of them got going again on their evolutionary development work. However, in one of the pilot project teams the process was interrupted. This study does not provide any answer to why this process was interrupted.

In this study, two parallel principles were found when implementing VBHC. One was the professionals’ voice and the other was the patients’ voice. The patients’ voice was described in Mishler’s metaphor as the ‘voice of the lifeworld’ [32]. The ‘voice of the lifeworld’ needs to be listened to not just in each encounter between physician and patient but also when managing healthcare. Participants in the project took the concept of value for the patient as their point of departure, but at the same time their professional perspective naturally permeated everything, i.e., ‘the voice of medicine’ [32]. Their profession-based understanding of what constitutes value for the patient mostly prevailed, especially when it came to deciding outcome measurements. However, over time and much due to the VBHC emphasis on explicitly asking patients what they considered valuable and important, realization dawned that health professionals do not always know what each specific patient finds valuable.

Patient representatives had their own ideas of what constituted valuable measurements, but these measurements were almost always considered impossible to execute. Instead, measurements were frequently chosen based upon ease of access to data. Porter and Lee warned against choosing indicators just because they are easy to measure. To avoid this pitfall they recommended three tiers of measuring, all focused on patient-related outcome. The first concerns achieved or retained health status, the second concerns the process of recovery and the last concerns the sustainability of health [5]. Each pilot project team had existing National Quality Registers for their group of patients, and these were the preferred choice and main source of data. As these registers have mainly been developed from interest in measuring medical outcome, ‘the voice of medicine’ was reinforced while ‘the voice of the lifeworld’ was weakened. Swedish National Quality Registers include aspects of disease management and some of them include patient-reported outcome measurements but to a lesser extent [33]. It is therefore important to raise the question: are National Quality Registers sufficient for measuring health outcomes when the intention is to create value for the patient? It would probably be important to develop local care quality registers in combination with National registers as has been done for one of the groups of patients. Another way to proceed might be to continue developing the National Quality Registers and to incorporate more of ‘the voice of the lifeworld’. If the old saying ‘what gets measured gets done’ is still valid, then this deserves further attention.

Consultants’ one-sided focus on measuring health outcomes was not always advantageous as improving health outcomes also presumed that processes were being developed. Defining clinical pathways for different groups of patients is important since it reduces variation and maximizes the outcomes [34]. The importance of developing the process of care to decrease lengths of hospital stay ensuring optimal patient experience have recently been stipulated in a study of primary hip and knee arthroplasties [35]. The development of any process requires basic shared understanding of the process among those working with or in it. Establishing such understanding requires leadership skills such as communication and motivation to get people involved step by step in developing the process [21]. Along with continued implementation of VBHC, the organizational culture also needs to further evolve towards a more patient-oriented way of working. This change calls for managers’ and employees’ efforts to participate in developmental work with reflections about what could be done jointly. Then new patterns of thinking and behaving can be developed in the organization [36]. It is important for managers to understand and respect that contradictory opinions can exist at the same time in the same organization [37] in order to facilitate the process of change.

The appreciation participants showed for the concept VBHC was not only connected to ‘value for the patient’ but was also related to the fact that they were tired of the focus on financial control that has been the main management trend for the last decades. Value for the patient was a driving force for participants but financial aspects are also of utmost importance to the concept of VBHC. As previously mentioned, value was defined as health outcomes attained per ‘dollar’ spent. An important factor behind the concept of VBHC was the need to solve the crisis of escalating costs in healthcare and therefore a new way of measuring cost was postulated in VBHC [5, 6]. Other explanations from the interviews related to the fact that the low interest in measuring costs might have been due to the hospital’s complicated IT system. The task of collecting particular data details of costs and not only aggregated data was too much for some participants. A further explanation of participants’ lesser degree of interest in measuring costs might be related to a reaction against the focus of NPM on internal control of financial aspects [28, 29]. However, VBHC is unlikely to become a replacement for NPM and its variants. Instead, a development towards management models with a mixture of governance according to VBHC and NPM principles might be expected [38]. VBHC could then be seen as supplying a broader perspective.

As already mentioned, Porter and colleagues stated that healthcare should be organized on the basis of patients’ medical conditions and care cycles [2–6]. That was not in focus when implementing VBHC at this hospital. From the start participants did not give this kind of issue much attention. This is understandable considering the intensive drive to show quick results regardless of the patients’ realities. However, over time and when developments became more internalized in daily work, the importance emerged of cooperation between different departments involved in the same patient journey. Some cooperation started during the study period, but additional challenges were experienced when changes involved crossing department and/or budgetary boundaries. The development of this cooperation in line with Porter’s and colleagues’ [2–6] ideas requires a very attentive upper management responding fast and accurately to new challenges as they arise – potentially reorganization of hospital departments and alignment of the budget system to patient processes. The heads of departments were central figures in the initiatives identified as including cooperation between departments, and it would be interesting to follow up this cooperation in future studies.

## Conclusion

An overall conclusion from this interview study was that participants appreciated the concept of VBHC by reason of its focus on value for the patients and measuring health outcomes, as opposed to previous experiences of management attention mostly measuring financial aspects of care. Working in line with VBHC was, mostly early in the process, experienced as justifying existing practices, i.e., in the best case to cure or alleviate suffering, and always to provide comfort and care. Although patient representatives were considered contributing valuable input towards finding relevant outcome measurements, their voices, i.e., ‘the voice of the lifeworld’ were weak compared to ‘the voice of medicine’. However, over time, when working in line with VBHC, participants sometimes found themselves being challenged as they needed to change their mindset about what patients’ themselves considered value.

The implementation of VBHC was not a straight linear process; the process moved forwards and backwards, sometimes with interruptions. The consultants’ support during the startup was appreciated. On the other hand, participants experienced the risk that their own future capability to manage the implementation process was diminished due to time pressure and the strict focus on outcome measurements. Only when the implementation process had proceeded, could the care process be further developed. These processes gave insights about the total complexity and the need of working together not just in participants’ own departments but also interdepartmentally, which also implied an awareness of the importance of working with care processes across boundaries. The need experienced by participants of working together with other professions and departments required a great deal of energy as breaking through organizational and administrative systems was hard. Healthcare organizations implementing management innovations such as VBHC therefore need to be aware of recognizing the intrinsic drive of healthcare practitioners, and to understand the complexity in healthcare itself as well as in the process of implementation.

## Implication

It is vital to estimate correctly the dimensions of the resources required by implementation work, not least regarding management and leadership, in order to facilitate the implementation of VBHC. Awareness of contradictory forces in action simultaneously, and adaptation to the effects of these forces, might simplify the process of implementation by facilitating communication and reflection on the developmental work in hand. It is also of utmost importance to reinforce the patients’ voice during the implementation work.

## Abbreviations

IP: Interview person declarations
VBHC: Value-based healthcare

## References

[1] Erichsen Andersson A, Baathe F, Wickström E, Nilsson K (2015). Understanding value-based healthcare – an interview study with project team members at a Swedish university hospital. JHA.

[2] Porter ME, Teisberg EO (2006). Redefining health care - creating value-based competition, 2006/06/06 edn.

[3] Porter ME, Teisberg EO (2007). How physicians can change the future of health care. Jama.

[4] Porter ME, Teisberg EO (2004). Redefining competition in health care. Harv Bus Rev.

[5] Porter M, Lee TH (2013). The strategy that will fix health care. Harv Bus Rev.

[6] Kaplan RS, Porter ME (2011). How to solve the cost crisis in health care. Harv Bus Rev.

[7] Fredriksson JJ, Ebbevi D, Savage C (2015). Pseudo-understanding: an analysis of the dilution of value in healthcare. BMJ Qual Saf.

[8] Lindgren P (2014). Reimbursement in health care. Models, effects, and recommendations (In Swedish).

[9] Wilson H, Gole J, Bharat M, Jitendra M (2016). Value based Healthcare. Advanc Manag.

[10] Bozic KJ, Wright JG (2012). Value-based healthcare and orthopaedic surgery: editorial comment. Clin Orthop Relat Res.

[11] Sallin K, Makdessi L, Sjogreen J (2015). The swedish society of medicine: value-based health care is loaded with problems, but also opportunities. Lakartidningen.

[12] Wohlin J, Aspelin P, Rehnqvist N, Dahlstrom T, Brommels M (2015). Value-based health care shifts the focus from care production to health. Lakartidningen.

[13] Nordenstrom J (2014). Value-based health care can improve health outcomes. Healthcare gets a new direction--the health personnel plays a key role. Lakartidningen.

[14] Flordal PA (2015). The birth, life and death of value-based health care. The last “pseudo-innovation”?. Lakartidningen.

[15] Kirkpatrick JR, Marks S, Slane M, Kim D, Cohen L, Cortelli M, Plate J, Perryman R, Zapas J (2015). Using value-based analysis to influence outcomes in complex surgical systems. J Am Coll Surg.

[16] McLaughlin N, Buxey F, Chaw K, Martin NA (2014). Value-based neurosurgery: the example of microvascular decompression surgery. J Neurol Surg.

[17] Øvretveit J, Andreen-Sachs M, Carlsson J, Gustafsson H, Hansson J, Keller C, Lofgren S, Mazzocato P, Tolf S, Brommels M (2012). Implementing organisation and management innovations in Swedish healthcare. J Health Organ Manag.

[18] Abdallah A (2014). Implementing quality initiatives in healthcare organizations: drivers and challenges. Int J Health Care Qual Assur.

[19] Hellström A, Lifvergren S, Gustavsson S, Gremyr I (2015). Adopting a management innovation in a professional organization. Bus Process Manag J.

[20] Hellström A, Lifvergren S, Quist J (2010). Process management in healthcare: investigating why it’s easier said than done. J Manuf Technol Manag.

[21] Nilsson K, Sandoff M (2015). Leading processes of patient care and treatment in hierarchical healthcare organizations in Sweden - process managers’ experiences. Leadersh Health Serv.

[22] Nilsson K, Sandoff M (2015). Managing processes of inpatient care and treatment. J Health Organ Manag.

[23] Wang Q, Li M, Zu H, Gao M, Cao C, Xia L (2013). A quantitative evaluation of health care system in US, China and Sweden. HealthMed.

[24] Swedish Association of Local Authorities and Regions (2008). The swedish healthcare system: how does it compare with other EU countries, the United States and norway?.

[25] Patton MQ (2002). Qualitative research & evaluation methods.

[26] Miles MB, Huberman AM (1994). Qualitative data analysis an expanded source-book.

[27] Ryan RM, Deci EL (2000). Self-determination theory and the facilitation of intrinsic motivation, social development, and well-being. Am Psychol.

[28] Christensen T, Lægreid P (2011). Complexity and hybrid public administration—theoretical and empirical challenges. Public Organ Rev.

[29] Hasselbladh H, Bejerot E, Gustafsson RÅ (2008). Beyond New public management - institutional transformation in swedish healthcare. [In swedish].

[30] Hansson SO (2014). Medical ethics and new public management in sweden. Camb Q Healthc Ethics.

[31] Sandoff M, Widell G (2015). Translating as response to paradoxes - when implementing HRM strategies in service organisations. German J Res Hum Resour Manage.

[32] Mishler EG (1984). The discourse of medicine. Dialectics of medical interviews.

[33] Emilsson L, Lindahl B, Köster K, Lambe M, Ludvigsson JF (2015). Review of 103 Swedish Healthcare Quality Registries. J Intern Med.

[34] Lawal AK, Rotter T, Kinsman L, Machotta A, Ronellenfitsch U, Scott SD, Goodridge D, Plishka C, Groot G (2016). What is a clinical pathway? Refinement of an operational definition to identify clinical pathway studies for a Cochrane systematic review. BMC Med.

[35] Cox J, Cormack C, Prendergast M, Celestino H, Willis S, Witteveen M (2016). Patient and provider experience with a new model of care for primary hip and knee arthroplasties. Int J Orthop Trauma Nurs.

[36] Suchman AL (2011). Organizations as machines, organizations as conversations Two core metaphors and their consequences. Med Care.

[37] Bååthe F, Ahlborg G, Edgren L, Lagström A, Nilsson K (2016). Uncovering paradoxes from physicians’ experiences of patient-centered ward-round. Leadersh Health Serv.

[38] Kragh Jespersen P (2005). Between profession and management. (In Danish).

[39] Act 2003:460 (Amended SFS 2008:192). The act of ethical trail of research concerning humans. Stockhom: Ministry of Education and Research. Date for promulgate 2003-06-05.

[40] World Medical Association: Declaration of Helsinki (2002). Ethical principles for medical research involving human subjects. Nurs Ethics.

